# The role of the Sheffield model on the minimum unit pricing of alcohol debate: the importance of a rhetorical perspective

**DOI:** 10.1332/174426415X14430986392944

**Published:** 2016-11

**Authors:** Srinivasa Vittal Katikireddi, Shona Hilton, Lyndal Bond

**Affiliations:** University of Glasgow, UK; Centre of Excellence in Intervention and Prevention Science

**Keywords:** minimum pricing, alcohol, rhetoric, econometric modelling

## Abstract

The minimum unit pricing (MUP) alcohol policy debate has been informed by the Sheffield model, a study which predicts impacts of different alcohol pricing policies. This paper explores the Sheffield model’s influences on the policy debate by drawing on 36 semi-structured interviews with policy actors who were involved in the policy debate. Although commissioned by policy makers, the model’s influence has been far broader than suggested by views of ‘rational’ policy making. While findings from the Sheffield model have been used in instrumental ways, they have arguably been more important in helping debate competing values underpinning policy goals.

## Introduction

Minimum unit pricing (MUP) of alcohol is a high-profile intervention with the potential to markedly improve population health and narrow health inequalities (Bambra et al, 2010; Anderson et al, 2009; Holmes et al, 2014). The policy debate in Scotland and the rest of the UK has been relatively unusual in public health terms in being heavily influenced by econometric modelling conducted at the University of Sheffield (Purshouse et al, 2010). Previous research has found that policy actors report modelling studies as being particularly helpful in informing policy decisions when evaluation-based evidence is unavailable, but how such modelling studies influence the policy process is less understood (Katikireddi et al, 2014a). In this paper, we explore the influences of the model on the MUP debate and the reasons for its prominence. To do so, we analyse data from interviews with policy actors, drawing upon relevant theories from the literature on the relationship between research and policy making.

We start by providing an introduction to the MUP policy context and then go on to provide an overview of the Sheffield model. We then introduce the theories informing our analysis and for the purposes of this paper, distinguish between theories that describe the different *influences* of research on the policy process and those that account for the different *processes* by which influence is achieved. After detailing our methodological approach, we describe the range of influences that the Sheffield model has had on the policy debate and identify reasons for these influences. In doing so, we discuss findings in relation to relevant literature. Finally, we review the utility of existing theoretical models in explaining the influences of the Sheffield model, and conclude by arguing that although the model has had important instrumental influences, its influence on the policy process can be better understood as a rhetorical tool.

## The minimum unit pricing policy context

Alcohol-related health and broader societal harms have been identified as a major priority by both Scottish and UK Governments (Scottish Government, 2008; HM Government, 2012). In contrast to other western European countries, both jurisdictions have experienced rapidly increasing rates of medical complications arising from alcohol use over the last four decades (Leon and McCambridge, 2006). Increasing affordability has been identified as an important reason for these increasing harms (Gillan and Macnaughton, 2007; Bennetts, 2008a; Academy of Medical Sciences, 2004), with systematic reviews demonstrating a consistent relationship between the cost of alcohol, rates of consumption and resultant harms (Wagenaar et al, 2009; Booth et al, 2008; Huaung, 2003). Historically, the price of alcohol has been influenced by changes in alcohol duty. However, the sale of alcohol products below the cost of duty alone indicates that such price increases may not always be passed on to consumers (Bennetts, 2008b; Black et al, 2011).

MUP of alcohol has emerged as a novel policy approach to reduce the affordability of alcohol in order to realise public health benefits, with its origins and the policy process by which it emerged having been studied elsewhere (Katikireddi et al, 2014c; Katikireddi, 2013; Katikireddi et al, 2014b; Katikireddi and Smith, 2014; Hawkins and Holden, 2013; McCambridge et al, 2013; Holden and Hawkins, 2013). While similar policies to increase the price of the cheapest alcohol exist elsewhere, most notably reference pricing in Canada (Stockwell et al, 2006), MUP differs in that it introduces a uniform minimum price based on alcohol content. Following the policy’s consideration at a workshop of public health experts (Gillan and Macnaughton, 2007), Scotland became the first country to pass legislation introducing MUP for alcohol, in May 2012 (Scottish Parliament, 2012) but this has yet to be implemented following legal challenges (BBC News, 2012). The policy, and in particular the role of evidence, has achieved a high profile amongst policy makers, mass media and alcohol-related industry actors (Wood et al, 2014; Hilton et al, 2014; Katikireddi and Hilton, 2014). Of note, evaluation-based evidence from Canada appears to have had less influence on the policy debate than econometric modelling (Katikireddi et al, 2014a).

## The Sheffield model

The School of Health and Related Research (ScHARR) at the University of Sheffield was initially commissioned by the UK Government’s Department of Health to carry out a systematic review of the relationship between the price and promotion of alcohol on consumption and harms (Booth et al, 2008). This work was extended to include a model of the impacts of potential policy options on health, crime and employment (hereafter referred to as the ‘Sheffield model’) (Brennan et al, 2008). The Sheffield model compared a variety of different policies including MUP (set at a range of different levels), a ban on below-cost sales, and a ban on off-licence promotions and increasing alcohol duty. These models have subsequently informed both Scottish Government policy deliberations (Robson, 2010; Health and Sport Committee, 2012) and the development of public health guidelines by the National Institute of Health and Clinical Excellence (NICE) (Purshouse et al, 2009). While each version of the model differs (particularly in response to the availability of new data), the fundamental principles of the modelling have remained the same.

The Sheffield model is essentially a deterministic causal model with two main components, an econometric component and an epidemiological component (Meier et al,2010). First, an econometric component (referred to as the ‘price-to-consumption’ model) relates policy interventions such as MUP, increases in alcohol duty, bans on below-cost sales and discount bans to price changes and hence consumption changes (Brennan et al, 2008). This component makes use of the principle of the price ‘elasticity’ of a product – a measure of the extent to which purchasing changes in response to price changes. The econometric part of the model uses UK or Scotland-based data to calculate ‘elasticities’ for different population subgroups (including by age, sex and drinker type, that is, moderate, hazardous or harmful consumption) and different types of product (beer, wine, spirits and alcopops). Allowing for heterogeneity enabled the Sheffield team to provide predictions as to how different groups of interest may respond to different policy interventions including MUP (Meier et al, 2010). In the epidemiological component, consumption changes were related to outcomes of interest (the ‘consumption-to-harm’ model) in a deterministic manner based on the principle of population attributable fractions. This allowed the Sheffield team to quantify harms prevented as a result of different policy options in terms of health, crime and economic benefits.

While detailed results are available elsewhere (Purshouse et al, 2010), an important finding has been that MUP represents a more targeted intervention than other pricing policy options, since it has the greatest impact on those drinking in the most harmful ways. This can be explained by the evidence that this population subgroup are most likely to consume the cheapest forms of alcohol (Black et al, 2011). In addition, the introduction of MUP is expected to prevent drinkers at the highest risk of harm from ‘trading down’ to cheaper products since they are already most likely to be consuming low-cost alcohol. The Sheffield model’s finding that MUP is a more targeted intervention for achieving public health benefits has been a key argument in its favour over increases in alcohol duty alone (Record and Day, 2009, Rice and Drummond, 2012).

## Theories relating evidence and policy

In common with international experience (for example, Lomas and Brown, 2009; Campbell et al, 2009; Lavis et al, 2002), interest around the importance of evidence-based and latterly evidence-informed policy has grown amongst both policy makers and researchers within the UK (Nutley et al, 2000; Solesbury, 2002; Sanderson, 2002). While there is research to suggest that statements arguing for ‘evidence-based policy’ have not been matched by instrumental evidence use (for example, Katikireddi et al, 2011, Baggott, 2011), the case of MUP raises issues for those advocating increased ‘use’ of evidence for policy making. Since MUP represents a relatively novel population-based intervention (albeit one where instructive comparable examples exist), concerns have been expressed that the lack of *a priori* evaluation-based evidence in such circumstances could be construed as a barrier to adoption (Smith et al, 2001). Advocates of evidence-informed policy have countered that a lack of evidence in such circumstances should not prevent innovation, but rather that available evidence should be marshalled to inform policy making and robust evaluation conducted (Macintyre et al, 2001). In the case of MUP, the policy debate has been prominently influenced by the Sheffield model.

A considerable number of theoretical models have been developed to describe, and to a lesser extent explain, the relationship between evidence and policy (Macintyre, 2012). Historically, evidence has been portrayed as influencing policy in an *instrumental* manner, and this perspective is still presented as the norm by the UK civil service, amongst others (Cabinet Office, 2003). Implicit in this view was the idea of policy making as a rational process that proceeded through a number of stages which allowed evidence to be drawn upon to identify problems and then determine the most appropriate option to pursue in response. Seen from this perspective, research may either be conducted prior to decision making or commissioned by decision makers to help in their deliberation (Weiss, 1979). However, instrumental use has been identified as less important than other influences of evidence (Nutley et al, 2000; Weiss, 1979; Haynes et al, 2011). *Conceptual* use suggests that evidence has helped policy makers think about an issue in a new way – in other words, research serves an enlightenment function. This form of influence can be difficult to trace, but it has been argued that this is often the most powerful influence evidence has in the long term (Weiss, 1977). A third broad category is *symbolic* use (Weiss, 1979). Weiss suggests that policy makers may, particularly for intractable areas of policy, draw upon evidence selectively to either support their position (political use) or to delay decision making (tactical use).

A separate emerging set of literature, emphasising the importance of *rhetoric*, provides an alternative perspective (Russell et al, 2008; Greenhalgh and Russell, 2006). Greenhalgh and colleagues (2006), building upon the work of political scientists (Stone, 1989), view policy making as ‘the authoritative exposition of values’, and argue that evidence therefore helps policy actors to deliberate on the resolution of competing values. Drawing upon Aristotle, they argue that there is a central role for rhetoric – the art of persuading others – which comprises:

three elements: logos – the argument itself; pathos – appeals to emotions (which might include beliefs, values, knowledge and imagination); and ethos – the credibility, legitimacy and authority that a speaker brings and develops over the course of the argument. (Russell et al, 2008)

They argue that the focus of the evidence-based policy movement has been on research evidence influencing policy making from a naive rationalist perspective, with less attention paid to the other two spheres. In contrast, they argue that rhetoric is a central part of policy making and evidence can, and indeed should, be used for rhetorical purposes, but they note the dearth of empirical research studying the role of rhetoric within the health field.

A considerable body of literature has developed to explain the lack of instrumental use of evidence, much of which has built on the idea that researchers and policy makers inhabit two different communities (Caplan, 1979). Importantly, Caplan does not just argue that the two communities do not come into contact with each other, but rather that a cultural gap exists. However, this model has been extensively critiqued since the distinction between the two communities is not clear-cut, particularly since many individuals move between the world of research and policy (Bartley, 1993). However, many current initiatives to improve research utilisation are underpinned by such a model and aim to bridge the gap between the research and policy communities (Lomas, 2007; Mitton et al, 2007). Knowledge transfer initiatives typically aim to push research findings to policy makers once research has been completed, thereby focusing on improving dissemination of findings (Lee and Garvin, 2003). In contrast, knowledge exchange initiatives emphasise the two-way processes between researchers and policy makers in jointly developing evidence, with researchers listening to and responding to the needs of end-users throughout the entire research process.

Informed by the literature above, this paper aims to explore the influences of the Sheffield model on the MUP policy debate and the reasons for its prominence, by drawing primarily on data from interviews with those involved in the policy process.

## Methods

One-to-one semi-structured confidential interviews (either face-to-face or telephone) were conducted as the primary form of data collection, given the need to obtain in-depth information on a potentially sensitive topic. Details of the methodology have been previously published (Katikireddi, 2013; Katikireddi and Hilton, 2014; Katikireddi et al, 2014b; Katikireddi et al, 2014c). These interviews were informed by two sets of document analysis – first, a narrative review of documents related to the development of MUP and second, a structured analysis of evidence submission documents by policy actors in response to a Scottish Parliamentary consultation (open from November 2009 to January 2010).

In total, 36 interviews were conducted between March 2012 and January 2013. While we note that categorisation of policy actors is problematic since movement between categories and dual membership is common, an indicative breakdown of interviewees is: eight academics, seven advocates, ten civil servants, six industry actors and five politicians. Participants were purposively selected to include a diverse range of positions with respect to support for MUP, relevance to Scottish and/or UK policy debates and a number of other dimensions (including political party for politicians, subsector within alcohol-related industries for industry actors, and department within the civil service for civil servants). Potential participants were initially identified from the two sets of document analysis mentioned above and supplemented by snowball sampling. In cases when a specific type of actor could not be interviewed, alternative participants were identified. Interviews continued until no major new themes emerged and participants from a diverse range of professional categories were obtained. The experiences of individual interviewees were taken into account during the analysis, including their role in the UK and/or Scottish debates, and referred to where relevant in the results. We also note that considerable heterogeneity lies within each category (for example, industry actors include alcohol producers, the licensed trade and supermarkets who all have different interests (Holden et al, 2012)) but for reasons of confidentiality, it is not possible to provide further details of the breakdown of participants beyond broad sector. However, diversity within each sector was sought and obtained.

Interviews were guided by a topic schedule that included questions on the evidence base around alcohol pricing policy, the role of the Sheffield model and views on the relationship between evidence and policy (including perceived differences between Scotland and the rest of the UK). Interviews typically lasted between 45 minutes and one hour. All interviews were conducted by the lead author (SVK). Interviewees were aware that the interviewer was trained as a medical doctor and often assumed the interviewer was familiar with epidemiology. Interviewees were also aware that the study was sponsored by the UK Medical Research Council and hence would tend to presume an interest in health. The research team has also been involved in developing plans for an evaluation of MUP and some interviewees were aware of this in advance of being interviewed (and this may have been a factor in influencing participation and the subsequent interview discussion).

The limited number of potential participants for this study increases the risk of interviewee identification and can also make recruitment difficult. In order to improve the potential for recruitment and the quality of data obtained, a tiered process was arranged for obtaining informed consent (Smith, 2008). Consent was obtained not just for participation but also for interview recording (obtained for nearly all cases), the use of quotations in publications and presentations (again available for most participants), and identification of the broad sector the participant was drawn from at the time of their involvement in MUP policy (that is, politician, civil servant, researcher, advocate and industry). Following the interview, transcripts were annotated by SVK to indicate sections not for quotation to help minimise the risk of disclosure. All participants were then provided with a copy of their transcript to review and were asked for any modifications that were required to ensure their anonymity (for example, indicating extra sections of the transcript that should be made not for quotation).

### Interview analysis

Interviews were transcribed and interview data were read repeatedly, coded thematically and re-coded to categorise emergent themes using NVivo 9. Coding initially proceeded inductively with descriptive codes being used to organise the data with the assistance of NVivo 9. Following this, data relevant to specific theories of the relationship between evidence and policy were coded using sets of ‘tree’ codes. New codes were used to capture further inductive themes in the data. The principle of the constant-comparative method was used to help identify explanations for patterns within the data, while also paying appropriate attention to contradictions and tensions within the data (Glaser and Strauss, 2009).

### Ethical approval

The study was reviewed and obtained ethical approval from the University of Glasgow’s College of Medicine and Veterinary Life Sciences Ethics Committee.

## Findings

### The many influences of the Sheffield model

In keeping with our expectations from document analysis, participants consistently and usually spontaneously highlighted the Sheffield model as having played a central role in the policy debate on MUP in both Scotland and the UK (although as identified elsewhere (Katikireddi et al, 2014c, Katikireddi and Smith, 2014), it did not appear to explain differences in policy outcomes between the two administrations). Indeed, many interviewees considered it the single most influential study, as suggested by one advocate:

Advocate: Well, certainly around minimum unit price we have, so we’ve looked at lots of, we’ve obviously looked at the Sheffield study, which has sort of become the [laughs],‘ the study’

Others echoed the opinion that the Sheffield model had become a real focus for debate with one civil servant referring to it as “the single most often referred to piece of work” in relation to MUP.

Despite the consensus on the Sheffield model’s importance, different (but not necessarily contradictory) views were expressed about how the Sheffield model influenced the policy debate, suggesting multiple ways of exerting influence. Some interviewees expressed the view that the work had been crucial in allowing MUP to emerge as a realistic policy option and suggested that, in its absence, there would have been a lack of confidence to pursue it:

Civil servant: Minimum unit pricing would never have flown if we hadn’t had something, you know, to kind of back it up. Frankly we were just, we were really lucky that Department of Health kind of commissioned ScHARR, you know, to do the work that they had done on sort of… the initial work that they did was on sort of comparing different types of affordability interventions. So, that kind of provided a starting point.

Academic: Well, I think the evidence around price has clearly been very influential, and then the modelled evidence of what effect the minimum unit price would have has clearly given people confidence that this proposal would have the desired effect. Not universally, but in terms of the balance of decision making.

It is worth noting here that both speakers highlight the importance of the Sheffield model as a means of persuasion to make MUP a credible policy intervention. However, there were clear indications of the importance of more instrumental use – particularly in two areas. First, the model was seen as helping to establish the principle and that the policy was targeted, that is, affected harmful and hazardous drinkers more than moderate drinkers:

Academic: And the fairness and reason behind a minimum price for a unit is kind of easily grasped, I think, at political levels as well. And then the modelling showing that this is going to have minimal impact on light drinkers and quite a big impact on heavy drinkers, it helps. So I think there’s an idea and some evidence and a way of presenting it that’s really got legs, and has been effective, it’s been easy for people to communicate and advance policy on the back of.

Here, the interviewee clearly describes an instrumental use of the Sheffield model, namely that a key finding from the model that those at highest risk from alcohol-related harms may be affected to a greater extent by the policy has been influential. However, they simultaneously emphasise the importance of the Sheffield model as a means of making a rhetorical argument.

The second area in which the Sheffield model exerted an instrumental influence on the MUP debate is in relation to the level that the minimum unit price should be set at:

Civil servant: So the Sheffield modelling is telling us that to get the impact we want, this is what you should set your price at, and 45p was the figure that was chosen the last time. Because we’ve got to satisfy European issues, because of barriers to trade and interference with the market ’cause it is a market intervention. So we’ve got to be able to justify that, and that’s where the modelling comes in.

The civil servant in the quotation above also highlights the importance of being able to present evidence to demonstrate the proportionality of the policy. This is necessary since MUP constitutes an intervention in an economic market, and must therefore represent a proportionate intervention in relation to its health objectives to be deemed legal under European trade law (Katikireddi and McLean, 2012). Therefore, the Sheffield model helps to provide the Scottish Government with a piece of evidence that can help justify their position in case of legal challenge (note that the above interview was carried out prior to both the passage of MUP legislation in Scotland and the instigation of legal challenges).

While the Sheffield model appears to have facilitated the emergence of MUP as a serious policy option and informed subsequent discussions about potential implementation of MUP, it would be misleading to suggest that policy actors merely responded to the emergence of this piece of evidence in a ‘rational’ manner. Indeed, many respondents suggested that the Sheffield model would often not influence the views of specific policy actors, one way or the other:

Advocate:… I could imagine that depending on what you want to hear, you’ll either see the modelling study as a very good piece of work or you’ll see it as a work of fiction. So I suspect it depends on your inherent belief. I’m not sure modelling studies sway people particularly, I think they just confirm what you already thought! It’s a bit cynical, but, you know, I can’t help but think, you know, if you don’t want to believe it, you can dismiss it as just being modelling.

In other words, interviewees suggested that policy actors frequently exhibited a confirmatory bias – perceiving the Sheffield model as a robust piece of research if already supportive of MUP but considering it merely a ‘modelling exercise’ if hostile. While this might at first seem contradictory to the high level of importance interviewees accorded to the Sheffield model, this is only the case if its impact is considered from an instrumental perspective. If instead a key influence of the Sheffield model has been as a rhetorical tool that highlights the health arguments for MUP (as opposed to other dimensions such as business considerations), then the Sheffield model can have simultaneously been influential while not necessarily influencing individual policy makers’ level of support.

Data from a respondent critical of MUP suggests that this may be the case:

Industry: That’s a difficult debate for us to be in, you know, arguing with experts, medical experts, about how many people are going to die or otherwise is a difficult place to be and yet the model is not infallible and changes dependent on what factors you put in… you’re then into quite a detailed argument about how the model works and what is and isn’t in it and where the factors are and yet the outward bit is about x number of people will die or not die. And it becomes quite a stark, it becomes quite an emotional debate. And that’s difficult for a retailer to engage in, that kind of debate.

Therefore the way the model worked to quantify harms helped to highlight the health aspects of the debate in an emotive manner (pathos) and strengthened the potential for the Sheffield model to serve as a rhetorical tool, that is, to present an argument in a favourable way to relevant audiences (such as the public, the mass media and politicians). This arguably helped health aspects of alcohol policy to be valued more, thus changing the way the policy issue is framed – known to be an important explanation for policy change (Riker, 1986), including in relation to MUP (Katikireddi et al, 2014b).

### Reasons for the Sheffield model becoming influential

A number of factors helped the Sheffield model to become influential in the policy process. First, the Sheffield model had clearly been designed to meet the needs of a particular policy situation. In the words of one interviewee:

Academic: So I think what’s been key has been the ability to answer the questions policy makers want answering and also to counter the criticisms that have been levelled at policies in the past. And part of that may have been that the Sheffield team had people who’ve been very good at going out and talking to people and actually getting those messages across. But I think also it is the way the model was designed was to answer policy questions.

The above quotation also highlights the importance of communication by the Sheffield team – thus providing some support for initiatives that seek to encourage researchers to disseminate findings across the ‘research-policy gap’. However, the quotation also suggests that it is not just the fact that the model answered a specific policy question, but also that the policy question was of interest to policy makers at the time. As noted earlier, the Sheffield model was specifically commissioned – first, by the Department of Health in England, then subsequently the Scottish Government and NICE. Therefore it appears that it is not only because the Sheffield model answered a question from a policy maker’s perspective but also that it was commissioned to answer a question already of interest:

Academic: Well, I think it [the Sheffield model] has a pivotal role, and I’m just reflecting now that it’s not just the evidence coming from outside that’s come to the policy, and affects the policy… my experience is that of all the research that’s ever done, it’s when Government asks and commissions research that it seems to have the most impact, that’s my experience. It’s uncanny, you know. When the Government asks, ‘can you do this research, can you model this’, and it’s done, then it’s fitted neatly into some existing process of decision making. The other stuff needs to go on, because it can feed in eventually to something like that.

Interview data showed that the original commissioning process with the Department of Health involved ongoing dialogue with a midpoint review to help ensure the findings would be of policy relevance. In addition, representatives of the Scottish Government were also in regular communication with Department of Health officials during this early period and articulating the Scottish interest in MUP early on. The collaborative approach between the Sheffield researchers and the civil servants commissioning the work therefore appeared to influence the development of the project, with the Sheffield team being guided by the civil servants as to what would be of policy relevance. One particularly good example of this exchange was the decision to quantify the extent of harms under different scenarios, as illustrated by one interviewee:

Academic: So the fact that the Sheffield Group won the tender, I think it was about five years ago, to model what would happen in different policy scenarios, looking at restricting advertising, marketing, cheap alcohol and so forth. And the evidence then was, it was a group that was very good at communicating with policy makers, ’cause they knew they wanted different scenarios modelled for them, you know, what would be the concrete effects? How many lives lost, how many hospital admissions prevented, economic costs saved, and so forth. They loved that. ‘And if we do this, what it’ll be, and if we do that’.

The origins of the Sheffield model therefore seem to relate far more closely to models of knowledge exchange than models of knowledge transfer, that is, engaging with policy makers throughout the research process. However, potentially in contrast to the knowledge exchange literature, the development of the Sheffield model does not appear to have served a merely instrumental or indeed political use (where the evidence was used merely to support a decision already taken). Instead, the preference for quantification of harms serves to reiterate the importance of considering the rhetorical functioning of the Sheffield model. The ability to quantify harms in such a way was appreciated by those involved in the policy process as very helpful, and indeed was noted by one interviewee to be a factor that helped the Sheffield team to be successful in their application.

While the collaborative approach between government officials and researchers helped create a piece of evidence that ultimately played a role in public health policy, some commentators did not consider this unproblematic. One respondent who was hostile to MUP did question the extent to which the Sheffield team’s work could be considered entirely impartial:

Academic: … I do think that when someone is hired to look at an issue where there is almost a presumption that the government is in favour of the policy then whoever you hire is more likely to come out with a supportive case. Just because they know why they’re being hired. But I think presenting something in as rosy a light as possible is a bit different than purposely biasing results. If you get me?

This interviewee, while being careful not to claim deliberate researcher misconduct, still questions researcher independence on the basis that the Sheffield team were commissioned to carry out their work. Industry representatives expressed similar concerns too. The fact that such a conflict of interest could be construed is noteworthy, since it suggests the perceived credibility (ethos) of the Sheffield team is questioned to help undermine the Sheffield model and in turn, the case for MUP. Relatedly, academics sometimes expressed discomfort that separate public health advocates were not always invited to key policy discussions, leaving them as the only health perspective presented and making it more difficult to present themselves as impartial.

### Building the reputation of the Sheffield model

The importance of reputation was a prominent theme amongst respondents and often related to the Sheffield model gaining influence on the policy process.

Civil servant: I do think evidence has played quite a big part in taking up minimum pricing …. The fact that Sheffield University had done quite a big review that was quite highly thought of had an impact.

Academic: So there is something very clear here about when a piece of evidence becomes recognised as a robust piece of science that can be relied on to give policy makers all of the information that they need, or the majority of the information they need to make about political decisions, that evidence can be very influential and that seems to be what we’ve seen here.

In both the quotations above, interviewees highlight the importance not just of the robustness of the Sheffield systematic review and model, but also that the work was *seen to be* well conducted. However, such a reputation was clearly not a given nor did it remain in a static condition. Rather, the reputation of the Sheffield team, particularly within the policy debate, was actively developed with the role of public debate being considered especially important. One politician explains this process eloquently:

Politician: … some of this is how we used the evidence in the legislative process, and for me that’s when the light went on above my head to say ‘I believe minimum pricing was right’. I read the conclusions of Sheffield, it’s very, very powerful, but I have to be confident that what Sheffield are saying is substantiated. And there’s a disengage between politician and expert at that level – you have to at some point trust in the experts that you asked to come up with these conclusions. So at the [Scottish Parliament’s Health and Sport] Committee what we had was two sets of experts. One for minimum pricing, one very lukewarm suggesting that it may not be worth the efforts, and they just had that debate in front of politicians and Sheffield came out with glowing colours, and that wasn’t a certainty. The reason they came out with glowing colours was because their evidence base was robust, because if it wasn’t robust the other guy would have exposed that. So that was the most powerful thing in terms of our committee and using an evidence base to say minimum pricing will work.

Here the interviewee suggests the public act of debate between researchers, which is the dialectical presentation of argument and counter-argument, has helped to position the Sheffield work as trustworthy. This *performative* element has in turn helped develop the reputation of the Sheffield model which, as seen above, helped to make the Sheffield model influential in policy circles.

### In(a)ccuracy of rhetoric

Interview data suggested the Sheffield model helped portray persuasive arguments in a manner which could be malleable to the political climate:

Interviewer: Just thinking about the evidence, you’ve mentioned that in England the driver for the introduction of a minimum unit price has probably come more from issues relating to binge drinking, especially amongst young people. Now, the modelling work actually tends to suggest that young people are not necessarily affected to as great an extent as some other groups for example. So, is there a potential mismatch between the evidence and how it’s being… ?

Academic: Well, I don’t know, I’m not sure I agree with your interpretation there because my understanding of it anyway is that young drinkers who are buying cheap alcohol are one of the principal parts of the modelling that I’ve seen. But assuming that we could maybe understand that same evidence differently, I don’t think it matters actually, because… and the reason I don’t think it matters is that, the young people focus provides the political hook which will pull everything through in its wake. So, even if that, if the evidence relating to youth drinking and the modelling is less… is less, it doesn’t, it’s less effective or whatever it is, I don’t think from a public health point of view that’s necessarily a problem, because it provides us with the political traction to bring in its wake a whole range of other beneficial public health effects.

The Sheffield model finds that young drinkers tend to consume a higher proportion of alcohol in the more expensive on-trade (such as pubs and nightclubs) and are therefore less affected than other groups by MUP (for example, a 3% fall in consumption for 18–24 years hazardous drinkers, compared to 6.9% for the overall population (Purshouse et al, 2010)) – a distinction not made by the above and other interviewees. This therefore suggests that although the Sheffield model did provide accurate arguments for the policy debate (as described earlier), the incorporation of the study into the policy debate resulted in the interpretation of some findings being altered. It is worth noting that this was linked by the respondent (who was highly knowledgeable on the evidence base) to the need for persuasive arguments that appear true (logos), rather than are demonstrably true in the policy process. In addition, the argument presented built on values that were politically more accepted, therefore facilitating the presentation of a persuasive case for MUP.

## Discussion

The Sheffield model has had an important impact on the MUP debate. While many health researchers and increasingly research funders aspire to increase the instrumental use of evidence on policy, we have found that even in the case of a directly commissioned piece of research, the influences on policy are complex. Findings from the Sheffield model had a direct influence on the policy process, with the model’s demonstration of MUP as a targeted intervention and its capacity to facilitate the comparison of different policy options (including the level at which to set a minimum unit price) particularly valued. However, at least as importantly, the Sheffield model served a rhetorical function. Its existence helped policy makers to present a rhetorical argument to a variety of audiences (including the media, public and politicians) that helped highlight the public health aspects of MUP. Rather than helping policy makers to achieve a predefined goal, the Sheffield model served to help advance public health interests by informing debates over contested values (Russell et al, 2008; Sanderson, 2006). Furthermore, policy makers’ awareness of the importance of persuasion helped shape the development of the Sheffield model in the first place.

A number of factors helped the Sheffield model attain an influential position in the policy debate. Consistent with existing theories that emphasise the importance of knowledge exchange (Contandriopoulos et al, 2010), the Sheffield model was developed through a collaborative approach between researchers and policy makers. Related to this collaborative approach, the Sheffield model demonstrated a close fit with the decision-making context, and was therefore seen as highly relevant by policy makers (Dobrow et al, 2006). These factors provide only a partial explanation for the Sheffield model’s success in achieving policy influence, however. An overarching reason for the Sheffield model’s influence was its potential to inform rhetorical debate. The model presented a range of arguments (logos), which appeared plausible, although not always accurately understood within policy circles, while also highlighting the health aspects of the policy debate (pathos). Its capacity to act as a successful rhetorical tool was not automatic but instead required ethos: the Sheffield model and its team had to actively develop a reputation as a credible source of expertise (Haynes et al, 2012). This involved undergoing ‘trials of strength’ whereby the Sheffield model / team had to undergo, and be seen to undergo, a process of argumentation before being viewed as legitimate (Latour, 1987).

Through a detailed analysis of the influences of a specific piece of evidence on a high-profile policy process, this paper has responded to calls for empirical research that adopts a rhetorical lens to studying the evidence-policy relationship (Greenhalgh and Russell, 2006; Russell et al, 2008). This perspective supplements the more widely used framework of Weiss (1979) and has been arguably more instructive for understanding the influence of a specific piece of evidence (rather than a body of evidence) in this case study. However, it should be noted that the rhetorical influence of evidence is underpinned by the roles of key policy actors and their strategic framing of causal stories (a point illustrated by Stevens (2007), for example) – these aspects have been investigated elsewhere in relation to MUP (Katikireddi et al, 2014b, Katikireddi et al, 2014c).

This study suggests that some tentative lessons for researchers who wish to improve the influence of their evidence on policy, an increasing concern across many countries including the UK, can be identified. First, the study confirms that ‘pull’ factors, where policy makers make requests from researchers, increase the likelihood of achieving research impact. Efforts to develop ongoing relationships with end-users through long-term knowledge exchange initiatives are therefore supported, but given the importance of various contextual factors described in relation to MUP (Katikireddi et al, 2014c), are unlikely to be sufficient by themselves. Second, research that produces arguments which can highlight specific values may increase potential for impact, and this could be more important in politically contested areas. Third, communication by researchers, especially when it happens in a public forum, can enhance the credibility of the study and thereby facilitate the achievement of impact.

In conclusion, this detailed analysis of the influences of a specific piece of evidence, within a high-profile policy debate, empirically illustrates the utility of a rhetorical perspective to analysing the influence of evidence on the policy process. While we do not wish to downplay the importance of instrumental use of evidence, especially in policy areas of low polarisation (Contandriopoulos et al, 2010), the analysis presented here demonstrates how rhetorical influences of evidence operate in the development of real-world public health policy. Rhetorical use of evidence can advance a health perspective to inform debates about the values that underpin public policy. Furthermore, the role of evidence in helping persuade audiences through highlighting specific values or goals (such as health outcomes) may influence the development of the evidence base. This interaction between the instrumental and rhetorical aspects of evidence highlights the need for a more integrated perception of research utilisation. Considering a rhetorical lens as fundamental differs from dominant approaches to the pursuit of healthy public policy (Bowman et al, 2012) and may better reflect the reality of the policy-making process.
